# Increasing Use of Advanced Practitioners: Strategizing for the Future

**Published:** 2014-01-01

**Authors:** Pamela Hallquist Viale

In 2009, the American Society of Clinical Oncology (ASCO) commissioned a study of collaborative practice arrangements using a national survey of oncology practices. Many of you may be familiar with this interesting report and its findings. ASCO projects that the demand for oncologists will outpace the supply of new oncologists in practice, with the capacity for patient visits rising a mere 14% (Towle et al., 2011). The demand for the services of an oncologist is expected to grow by an astounding 48% by 2020 (Towle et al., 2011). It is not difficult to see that while patient demand and need will continue to rise, a significant shortage of oncologists will occur in the near future.

## Growing Role of NPs and PAs: Perspectives in Oncology AND Primary Care

The ASCO study demonstrated several significant observations. It is important to note that oncology patients are aware of when an advanced practitioner (AP) provides their care and that they are very satisfied with that care. Collaborative practice arrangements are well accepted, and practices surveyed showed a 19% increase in productivity as measured by increased numbers of patient encounters. Both physicians and APs are highly satisfied with and strongly supportive of the collaborative practice model (Towle et al., 2011).

These findings are in contrast to the survey published by Donelan and colleagues on expanded roles of practitioners in primary care (Donelan, DesRoches, Dittus, & Buerhaus, 2013). In a study of 972 clinicians, they noted that more than 66% of the physicians surveyed thought that physicians provided higher-quality patient exams and consultations than nurse practitioners (NPs); most of the NPs disagreed. And although 77% of the NPs thought their roles would reduce the costs of care, only 31% of the physicians agreed, and 10% of them believed that expanded use of NPs would increase costs.

The primary care physicians and NPs (72.5% and 90.5%, respectively) in the Donelan survey agreed that increased numbers of NPs would help provide faster access to care. However, 31% of the physicians thought that expanded use of NPs would make safety problems worse, in contrast to the 21% of physicians who predicted improved levels of safety for their patients.

## More Research Needed

The primary care survey concluded that physicians and practitioners do not agree about their roles in primary care and that expansion of the role of the NP is controversial. The view provided by the ASCO Collaborative Practice Study strongly supports the expanded use of APs as a positive strategy to improve care and meet the projected increased demands of oncology care in the future.

More research is clearly needed to further evaluate the current roles of the AP and potential benefits associated with their growing role. I would also like to see more research done in different practice settings to further determine the effectiveness of the AP. Although traditional use of NPs and physician assistants (PAs) has been rooted in the community or outpatient setting, APs have been utilized in acute care settings as well.

A recently published study examined the use of PAs in caring for patients with acute myelogenous leukemia in the critical care setting (Glotzbecker et al., 2013). Although these were very sick patients, retrospective data demonstrated equivalent mortality, with a decrease in length of stay, readmission rates, and consults for those patients cared for by PAs. These outcomes suggest that APs are capable of providing appropriate and even improved care to critically ill patients in an acute care setting. It is clear that we have more work to do to continue to validate the roles of the NP and PA in both the primary care and oncology settings.

## JADPRO Live

We are getting very close to our first on-site educational conference: JADPRO Live! The sunny city of St. Petersburg, Florida, is the location for this first AP conference, offering up to 12 continuing education units. A renowned faculty and exciting agenda promise to provide 3 days of critical updates in oncology, with opportunities to network and discuss the issues facing APs today. Don’t miss this opportunity to hear major oncology leaders discuss their perspectives on the role of the AP in oncology care. If you haven’t already registered, please do so. If you are already registered, I look forward to seeing you there.
